# Associations between air pollution, intracellular-to-extracellular water distribution, and obstructive sleep apnea manifestations

**DOI:** 10.3389/fpubh.2023.1175203

**Published:** 2023-06-15

**Authors:** Cheng-Yu Tsai, Huei-Tyng Huang, Ming Liu, Wun-Hao Cheng, Wen-Hua Hsu, Yi-Chun Kuan, Arnab Majumdar, Kang-Yun Lee, Po-Hao Feng, Chien-Hua Tseng, Kuan-Yuan Chen, Jiunn-Horng Kang, Hsin-Chien Lee, Cheng-Jung Wu, Wen-Te Liu

**Affiliations:** ^1^Department of Civil and Environmental Engineering, Imperial College London, London, United Kingdom; ^2^Division of Pulmonary Medicine, Department of Internal Medicine, Taipei Medical University-Shuang Ho Hospital, New Taipei City, Taiwan; ^3^Department of Medical Physics and Biomedical Engineering, University College London, London, United Kingdom; ^4^Department of Biology, University of Oxford, Oxfordshire, United Kingdom; ^5^School of Respiratory Therapy, College of Medicine, Taipei Medical University, Taipei, Taiwan; ^6^Respiratory Therapy, Division of Pulmonary Medicine, Department of Internal Medicine, Taipei Medical University-Wan Fang Hospital, Taipei, Taiwan; ^7^Sleep Center, Shuang Ho Hospital, Taipei Medical University, New Taipei City, Taiwan; ^8^Department of Neurology, Taipei Medical University-Shuang Ho Hospital, New Taipei City, Taiwan; ^9^Department of Neurology, School of Medicine, College of Medicine, Taipei Medical University, Taipei, Taiwan; ^10^Taipei Neuroscience Institute, Taipei Medical University, Taipei, Taiwan; ^11^Research Center of Artificial Intelligence in Medicine, Taipei Medical University, Taipei, Taiwan; ^12^Graduate Institute of Nanomedicine and Medical Engineering, College of Biomedical Engineering, Taipei Medical University, Taipei, Taiwan; ^13^Department of Psychiatry, Taipei Medical University Hospital, Taipei, Taiwan; ^14^Department of Otolaryngology, Taipei Medical University-Shuang Ho Hospital, New Taipei City, Taiwan

**Keywords:** air pollution, obstructive sleep apnea, PM_2.5_, PM_10_, intra-to-extracellular body water distribution

## Abstract

**Background:**

Exposure to air pollution may be a risk factor for obstructive sleep apnea (OSA) because air pollution may alter body water distribution and aggravate OSA manifestations.

**Objectives:**

This study aimed to investigate the mediating effects of air pollution on the exacerbation of OSA severity through body water distribution.

**Methods:**

This retrospective study analyzed body composition and polysomnographic data collected from a sleep center in Northern Taiwan. Air pollution exposure was estimated using an adjusted nearest method, registered residential addresses, and data from the databases of government air quality motioning stations. Next, regression models were employed to determine the associations between estimated air pollution exposure levels (exposure for 1, 3, 6, and 12 months), OSA manifestations (sleep-disordered breathing indices and respiratory event duration), and body fluid parameters (total body water and body water distribution). The association between air pollution and OSA risk was determined.

**Results:**

Significant associations between OSA manifestations and short-term (1 month) exposure to PM_2.5_ and PM_10_ were identified. Similarly, significant associations were identified among total body water and body water distribution (intracellular-to-extracellular body water distribution), short-term (1 month) exposure to PM_2.5_ and PM_10_, and medium-term (3 months) exposure to PM_10_. Body water distribution might be a mediator that aggravates OSA manifestations, and short-term exposure to PM_2.5_ and PM_10_ may be a risk factor for OSA.

**Conclusion:**

Because exposure to PM_2.5_ and PM_10_ may be a risk factor for OSA that exacerbates OSA manifestations and exposure to particulate pollutants may affect OSA manifestations or alter body water distribution to affect OSA manifestations, mitigating exposure to particulate pollutants may improve OSA manifestations and reduce the risk of OSA. Furthermore, this study elucidated the potential mechanisms underlying the relationship between air pollution, body fluid parameters, and OSA severity.

## 1. Introduction

Obstructive sleep apnea (OSA) is a common sleep-related breathing disorder. Approximately 1 billion individuals aged between 30 and 65 years have OSA, making it a major global health concern (1). The prevalence of OSA increased by approximately 30% from 1990 to 2010, affecting 17% of men and 34% of women aged between 30 and 70 years in the United States (2, 3). OSA is caused by the obstruction of the upper respiratory tract (including partial [hypopnea] and complete [apnea] occlusion), which leads to airflow restriction episodes and, consequently, oxygen desaturation (4). Various risk factors for OSA have been identified, including changes in body water distribution and air pollution (5). However, the interactions between these two factors remain unclear. Determining the associations between these factors and OSA and investigating the synergistic effects of these factors on OSA can assist in reducing the increasing prevalence of OSA.

Polysomnography (PSG) is mainly used to evaluate OSA severity (apnea–hypopnea index [AHI]), oxygen desaturation level (oxygen desaturation index [ODI]), and sleep arousal occurrence frequency (arousal index [ArI]) in patients with OSA, and these parameters are affected by air pollutants. For example, a review of 15 published studies reported that nitrogen dioxide (NO_2_), ozone (O_3_), and particulate matter (PM) were associated with increased AHI, ArI, and ODI values (6). Another study indicated that an interquartile range (IQR) increase in 4-year mean exposure to ambient PM, including particles with diameters of ≤2.5 μm (PM_2.5_) and ≤ 10 μm (PM_10_), was associated with an increased risk of sleep disorders, with the odds ratio (OR) being 1.47 (95% confidence interval [CI] = 1.34–1.62) for PM_2.5_ and 1.17 (95% CI = 1.02–1.34) for PM_10_ (7). Similarly, exposure to high concentrations of air pollutants (PM_2.5_, PM_10_, and NO_2_) in the short term (1 month) was associated with an increased occurrence of arousal and alterations in sleep architecture in patients with OSA (8). The aforementioned findings indicate that air pollutants may aggravate OSA manifestations and increase OSA severity. In addition, a study reported that respiratory event duration can indicate arousal ability or even predict mortality. This is because this duration can reflect levels of hypoxemia, hypercapnia, and end-inspiratory effort, which are key physiological stressors for respiratory distress (9). To date, no study has investigated the association between exposure to air pollutants and respiratory event duration. However, understanding such an association can provide insight into underlying pathophysiological mechanisms.

Total body water (TBW) and body water distribution (i.e., the balance between intracellular and extracellular water [IE distribution]) can affect OSA manifestations. A review reported that fluid overload can aggravate OSA manifestations; thus, interventions aimed at reducing body fluid accumulation can substantially attenuate OSA severity (10). Another study analyzed data on body fluids from multiple poststroke cohorts with OSA, and it reported that reducing nocturnal fluid shift alleviated OSA severity (11). Air pollution can affect TBW and IE distribution and thereby indirectly exacerbate OSA severity. For example, a review reported that the prolonged absorption of air pollutants can lead to the inflammation of the airway mucosa and to changes in the osmotic pressure of cell membranes. These changes can alter the volumes of intracellular water (ICW) and extracellular water (ECW) over time, leading to upper airway edema and increased OSA severity (12). Another review indicated that PMs containing various metals and silicate-derived constituents can significantly increase the risk of edema involving the alveoli and induce respiratory irritation because of the PMs’ cytotoxicity (13). Another study reported that exposure to fine air pollutants triggers oxidation. This phenomenon increases the oxidative stress on cells and, consequently, affects the osmotic pressure of human alveolar epithelial cell membranes, which can lead to changes in the ICW–ECW balance (14). The aforementioned findings suggest partial associations between exposure to air pollutants, body water distribution, and OSA. However, further exploration is required to clarify the relationships among these factors.

The present retrospective study investigated the associations among exposure to air pollutants, body water distribution, and OSA manifestations (i.e., AHI, ODI, ArI, and respiratory event duration); the mediating effects of these variables were also explored. In addition, the present study investigated the effect of air pollution on OSA risk. First, we obtained the data of healthy individuals and patients with OSA from a sleep center in northern Taiwan. Subsequently, on the basis of the residential addresses of the patients, we estimated their exposure to various air pollutants and investigated the relationships among the air pollution estimates, body water distribution, and PSG parameters. The findings of this study can provide insight into the associations among air pollution, alterations in body water distribution, and OSA manifestations.

## 2. Materials and methods

### 2.1. Ethics statement

This study and its protocols were approved by the Joint Institutional Review Board of Taipei Medical University (approval number: N202212067).

### 2.2. Included patients

This study retrospectively analyzed the data of patients who underwent treatment between July 2019 and February 2022 at a sleep center (Taipei Medical University-Shuang Ho Hospital, New Taipei City, Taiwan). Patients were included if they (1) underwent a complete PSG examination (total recording time of >6 h), (2) were aged between 18 and 80 years, (3) were not undergoing any treatment for OSA (surgery or wearing of noninvasive devices), (4) were not regularly using hypnotics or psychotropics, and (5) were not given a diagnosis of central nervous system disorders (e.g., stroke, brain tumor, and epilepsy) or lung diseases (e.g., chronic obstructive pulmonary disease and lung cancer). We obtained the clinical records of eligible patients to acquire their baseline information (age and sex), anthropometric measurements (body mass index [BMI], neck and waist circumferences, data on body composition and body water distribution), and residential addresses. All derived data were further analyzed.

### 2.3. Anthropometric measures

Anthropometric measures, which are routinely obtained before PSG is performed, were obtained from the aforementioned medical database. Waist and neck circumferences were measured using a tape measure by PSG technologists. Data on body composition and body fluid distribution were obtained using the Tanita MC-780 system (Tanita, Tokyo, Japan). The measurement procedure was as follows. Patients were required to undergo bioelectrical impedance analysis prior to their PSG. All patients were asked to fast for at least 3 h and to evacuate their bladder before they were measured. Subsequently, each patient was asked to stand still with their feet shoulder width apart on the machine platform while holding induction metal handles with both arms placed straight down. After approximately 10 s, the system automatically acquired measurements of the patient’s visceral fat level, total body fat percentage, and muscle mass percentage. In addition, data on body fluid, including TBW and body water distribution (the percentages for ICW and ECW), were collected. To determine whether body water stayed within cells and tissue (intracellular) or flowed to spaces outside of cells (extracellular), we calculated the IE distribution by applying the following formula: ICW/ECW × 100. These retrospective measurements were used in a subsequent statistical analysis.

### 2.4. PSG parameters

The sleep center used three forms of lab-based PSG equipment: an Embla N7000 (ResMed, San Diego, CA, USA), an Embletta MPR (Natus Medical, Pleasanton, CA, USA), and a Nox-A1 (Nox Medical, Alpharetta, GA, USA). In addition, two types of scoring systems, namely the RemLogic software (version 3.41; Embla Systems, Thornton, CO, USA) and Noxturnal system (version 6.2.2; Nox Medical), were used in conjunction with various lab-based PSG equipment. All scoring processes were performed by certified PSG technologists in accordance with the scoring manual published in 2017 by the American Academy of Sleep Medicine (15). To ensure scoring reliability and minimize individual bias, the scoring outcomes were reviewed by a principal scoring technologist and an independent technologist, and any inconsistency in their findings was discussed until a consensus was reached. Respiratory events were scored using specific scoring criteria; apnea was defined as a decrease of ≥90% in the oronasal thermistor signal, and hypopnea was defined as a decrease of ≥30% in the nasal prong pressure signal combined with the occurrence of oxygen desaturation (≥3%) or arousal. Event duration and total oxygen desaturation level (≥3%) were determined on the basis of this scoring system. Arousal was defined as the presence of a brainwave alteration that lasted for at least 3 s with high-frequency patterns (e.g., alpha wave [8–12 Hz], theta wave [4–8 Hz], and high-frequency wave [>16 Hz]; the sleep spindle is excluded) and was preceded by stable sleep (≥10 s). After the completion of respiratory event scoring, AHI, ODI, and ArI were calculated by dividing the event score by the duration of sleep. On the basis of the obtained AHI values, OSA severity was categorized as normal (AHI < 5 events/h), mild (AHI = 5–15 events/h), moderate (AHI = 15–30 events/h), and severe (AHI ≥ 30 events/h) (16).

### 2.5. Estimation of exposure to air pollutants

On the basis of the residential addresses of the patients, we acquired hourly monitoring data on temperature, humidity, and air pollutant concentration levels from multiple government-maintained air quality stations. A total of 19 stations in Taiwan are subsidized and managed by Taiwan’s Environmental Protection Administration. We used data from 16 stations and excluded those from the other 3 stations because 1 was located in a national park and the other 2 were background stations. To estimate exposure to air pollutants, we used an adjusted method that was proposed in another study and considered data from the nearest station (17). In the present study, only stations located within 3 km of a patient’s residential address were selected. Next, the weights of the selected stations were calculated on the basis of their distances from the residential address of a patient, and the weighted mean of the daily pollutant concentrations recorded at these stations was calculated (Supplementary Figure S1). Using data from a single nearest station (i.e., a single data source) can result in imprecise estimates if the station is located far away from the residence of a patient. Thus, this study used data from multiple stations to overcome this problem. For air pollutant type, the present study obtained the median concentrations and IQRs for PM_10_, PM_2.5_, CO, neutral oxide, NO_2_, SO_2_, and O_3_. For each patient, data pertaining to their exposure at 1, 3, 6, and 12 months prior to their PSG were obtained. These exposure data were used to determine the short-term (1 month), medium-term (3 and 6 months), and long-term (12 months) effects of exposure.

### 2.6. Statistical analysis

First, we used multiple linear regression models to investigate the associations between air pollution, body water distribution, and OSA manifestations. We used body water distribution parameters as mediators and employed multiple linear regression models to investigate the synergistic effects of air pollution and body water distribution on OSA severity. This analysis was conducted to determine whether the independent variable (air pollution) affected the intermediary variable (body water distribution) to partially or fully affect the dependent variable (OSA manifestations). We used multivariable logistic regression models to calculate the odds ratio for normal individuals (AHI < 5 events/h) and patients with OSA (AHI ≥ 5 events/h) to investigate the effects of exposure to air pollutants; IQR changes were used to represent individual pollution exposure. Furthermore, to investigate the effects of exposure to ambient air pollutants on the sleep parameters for different sexes, the present study conducted a subgroup analysis. All statistical analyses were performed using SPSS (version 20.0; IBM, Armonk, NY, United States). The level of significance was set at *p* < 0.05.

## 3. Results

### 3.1. Patients’ basic characteristics, sleep parameters, and exposure level to air pollutants

This study retrospectively collected the data of 2,906 patients. Tables 1, 2 summarize their demographic data and sleep parameters, respectively. Table 3 presents the details of these patients’ exposure to air pollutants. The mean age of the patients was 47.44 years, and 66.28% of the patients were men. The patients had a mean BMI of 26.7 kg/m^2^, mean neck circumference of 37.57 cm, mean waist circumference of 91.43 cm, mean visceral fat level score of 11.62, mean fat percentage of 28.67%, and mean muscle mass percentage of 17.85%. Their distribution was 49.84% ± 5.54% for TBW, 58.56% ± 2.57% for ICW, 41.44% ± 2.57% for ECW, and 142.25 ± 14.8 for IE. For OSA severity, 316 (10.87%) of the patients were categorized as normal individuals, 658 (22.64%) had mild OSA, 754 (25.95%) had moderate OSA, and 1,178 (40.54%) had severe OSA. For sleep quality, the patients had an ODI of 24.63 ± 24.39 events/h, an AHI of 30.34 ± 24.47 events/h, and an ArI of 21.72 ± 14.81 events/h; furthermore, their respiratory event duration was 25.29 ± 5.85 s. For the patients’ level of exposure to air pollutants and their background atmospheric information, their short-term IQR (1 month) was 3.39 μg/m^3^ for PM_10_ and 2.24 μg/m^3^ for PM_2.5_, and their medium-term IQRs were 2.41 (3 months) and 2.28 μg/m^3^ (6 months) for PM_10_ and 1.74 (3 months) and 1.47 μg/m^3^ (6 months) for PM_2.5_. The IQRs for air pollutants decreased over time, whereas the median values for air pollutants remained mostly stable over time across all measurement time points.

**Table 1:** Demographic and body profiles of study population (*N* = 2,906).

Categorical variable	*n*/Mean	%/SD
Age (y)	47.44	13.13
Sex (*n*, %)		
Male/Female	1926/980	66.28/33.72
Body profile		
BMI (kg/m^2^)	26.7	4.8
Neck circumference (cm)	37.57	4.73
Waist circumference (cm)	91.43	12.3
Visceral fat level (score)	11.62	4.82
Fat percentage (%)	28.47	8.73
Muscle mass percentage (%)	17.85	4.54
Body water parameters		
TBW (%)	49.84	5.54
ICW (%)	58.56	2.57
ECW% (%)	41.44	2.57
IE distribution	142.25	14.8
OSA severity (*n*, %)		
Normal	316	10.87
Mild	658	22.64
Moderate	754	25.95
Severe	1,178	40.54

SD, standard deviation; BMI, body mass index; TBW, total body water; ICW, intracellular water; ECW, extracellular water; IE distribution, intracellular-to-extracellular water distribution; OSA, obstructive sleep apnea.

IE distribution = ICW (%)/ECW (%) × 100.

**Table 2:** Sleep parameters obtained from polysomnography results of study population (*N* = 2,906).

Categorical variables	Mean	SD
Sleep architecture		
Sleep efficiency (%)	77.99	11.31
Wake (% of SPT)	15.96	10.34
NREM (% of SPT)	71.72	9.4
REM (% of SPT)	12.29	6.04
WASO (min)	54.35	35.8
TST (min)	285.71	42.72
Sleep quality index (events/h)		
ODI	24.63	24.39
AHI (events/h)	30.34	24.47
ArI	21.72	14.81
Snoring index	223.76	218.01
Respiratory event duration (s)	25.29	5.85
Oximetry parameter (%)		
Mean SpO_2_	94.91	2.31
Lowest SpO_2_	82.98	8.87

SPT, sleep period of time; NREM, nonrapid eye movement; REM, rapid eye movement; WASO, wake time after sleep onset; TST total sleep time; ODI, oxygen desaturation index; AHI, apnea–hypopnea index; ArI, arousal index; SpO_2_, oxygen saturation (measured using pulse oximetry).

ODI is defined as the cumulative frequency of desaturation episodes (≥3%) occurring per hour.

**Table 3:** Exposure to air pollution and background conditions of study population.

Categorical variables	Median (IQR)			
	1 month	3 months	6 months	12 months
Air pollutants				
PM_10_ (μg/m^3^)	22.9 (3.39)	22.55 (2.41)	23.81 (2.28)	24.77 (1.7)
PM_2.5_ (μg/m^3^)	11.98 (2.24)	12.01 (1.74)	12.39 (1.47)	12.91 (0.63)
NO_2_ (ppb)	14.45 (2.18)	14.34 (1.96)	14.39 (1.6)	15.04 (1.11)
SO_2_ (ppb)	1.83 (0.27)	1.85 (0.27)	1.86 (0.22)	1.92 (0.21)
O_3_ (ppb)	28.64 (4.07)	28.82 (3.35)	28.5 (1.32)	28.9 (0.6)
Background	Mean (SD)			
Ambient temperature (°C)	23.57 (4.65)	23.85 (4.36)	24.25 (3.04)	24.08 (0.32)
Relative humidity (%)	73.51 (4.71)	73.36 (3.83)	73.06 (2.6)	72.92 (1.52)

IQR, interquartile range; PM_10_, particulate matter with an aerodynamic diameter of ≤ 10 μm; PM_2.5_, particulate matter with an aerodynamic diameter of ≤ 2.5 μm; NO_2_, nitrogen dioxide; SO_2_, sulfur dioxide; O_3_, ozone.

Data are expressed as medians (interquartile ranges).

### 3.2. Associations between air pollutants and sleep quality indices

The present study analyzed the associations between sleep quality indices and exposure to air pollutants in the short, medium, and long terms (Table 4). Increased IQRs for PM_10_ and PM_2.5_ in the short term (1 month) were significantly associated with increased AHI, ODI, and ArI levels after adjustments for age, sex, BMI, temperature, and relative humidity. Furthermore, an increased IQR for PM_10_ was significantly associated with a longer respiratory event duration (0.32 s, 95% CI = 0.08–0.55 s, *p* < 0.01). An increased IQR for PM_2.5_ was significantly associated with a longer respiratory event duration (1.18 s, 95% CI = 0.94–1.42 s, *p* < 0.01). These results indicate that a high level of exposure to PM_10_ and PM_2.5_ in the short term aggravated OSA manifestations and affected sleep quality in terms of the frequency and duration of respiratory events. Next, a subgroup analysis of sex-stratified data was conducted to investigate the association between air pollution and sleep quality indices. The results are presented in Supplementary Tables S1, S2. For male patients, an increased IQR for short-term exposure to PM_2.5_ and PM_10_ significantly influenced AHI, ODI, and ArI levels after adjustments for age, BMI, temperature, and relative humidity. For female patients, the results after adjustments for age, sex, BMI, temperature, and relative humidity indicated that an increased IQR for PM_10_ significantly influenced their AHI and respiratory event duration, whereas an increased IQR for PM_2.5_ significantly influenced their ODI and respiratory event duration.

**Table 4:** Associations among sleep quality indices and interquartile range alterations in short-, medium-, and long-term exposure to air pollution.

Categorical variables	Beta Coefficient (95% Confidence Interval)			
	1 month	3 months	6 months	12 months
AHI (events/h)				
PM_10_ (μg/m^3^)	1.92 (1.06 to 2.79)**	−0.37 (−1.34 to 0.61)	0.41 (−0.33 to 1.15)	−0.31 (−1.05 to 0.43)
PM_2.5_ (μg/m^3^)	2.99 (2.1 to 3.89)**	0.31 (−0.42 to 1.05)	0.31 (−0.43 to 1.04)	−0.0 (−0.74 to 0.73)
NO_2_ (ppb)	0.09 (−0.65 to 0.82)	−0.55 (−1.29 to 0.18)	0.08 (−0.66 to 0.82)	−0.35 (−1.08 to 0.39)
SO_2_ (ppb)	−0.24 (−0.97 to 0.5)	0.32 (−0.42 to 1.05)	0.14 (−0.59 to 0.88)	−0.68 (−1.42 to 0.06)
O_3_ (ppb)	0.62 (−0.22 to 1.46)	−0.0 (−0.74 to 0.73)	0.51 (−0.23 to 1.24)	0.29 (−0.44 to 1.03)
ODI (events/h)				
PM_10_ (μg/m^3^)	2.02 (1.17 to 2.88)**	−0.09 (−1.06 to 0.87)	0.26 (−0.47 to 0.99)	−0.04 (−0.77 to 0.7)
PM_2.5_ (μg/m^3^)	3.29 (2.41 to 4.18) **	0.25 (−0.48 to 0.98)	0.21 (−0.52 to 0.94)	0.17 (−0.56 to 0.9)
NO_2_ (ppb)	0.02 (−0.71 to 0.75)	−0.51 (−1.24 to 0.21)	−0.01 (−0.74 to 0.72)	−0.06 (−0.79 to 0.67)
SO_2_ (ppb)	−0.48 (−1.21 to 0.25)	0.42 (−0.31 to 1.15)	−0.04 (−0.76 to 0.69)	−0.67 (−1.41 to 0.06)
O_3_ (ppb)	0.63 (−0.2 to 1.47)	−0.04 (−0.77 to 0.69)	0.35 (−0.38 to 1.08)	0.11 (−0.62 to 0.84)
ArI (events/h)				
PM_10_ (μg/m^3^)	0.92 (0.32 to 1.52)**	−0.68 (−1.35 to −0.01)	0.47 (−0.03 to 0.98)	−0.24 (−0.75 to 0.26)
PM_2.5_ (μg/m^3^)	1.23 (0.6 to 1.85)**	0.08 (−0.43 to 0.58)	0.46 (−0.05 to 0.96)	−0.15 (−0.66 to 0.35)
NO_2_ (ppb)	−0.09 (−0.61 to 0.42)	−0.43 (−0.94 to 0.07)	0.24 (−0.27 to 0.75)	−0.25 (−0.75 to 0.26)
SO_2_ (ppb)	−0.11 (−0.62 to 0.4)	0.37 (−0.14 to 0.87)	0.16 (−0.34 to 0.67)	0.06 (−0.45 to 0.57)
O_3_ (ppb)	−0.53 (−1.11 to 0.06)	0.16 (−0.35 to 0.66)	0.41 (−0.1 to 0.91)	0.11 (−0.39 to 0.62)
Respiratory event duration (s)				
PM_10_ (μg/m^3^)	0.32 (0.08 to 0.55)*	0.08 (−0.19 to 0.34)	−0.1 (−0.3 to 0.1)	0.03 (−0.17 to 0.23)
PM_2.5_ (μg/m^3^)	1.18 (0.94 to 1.42)**	−0.1 (−0.3 to 0.1)	−0.12 (−0.31 to 0.08)	−0.18 (−0.38 to 0.02)
NO_2_ (ppb)	0.0 (−0.2 to 0.2)	−0.04 (−0.24 to 0.16)	−0.1 (−0.3 to 0.1)	−0.12 (−0.32 to 0.08)
SO_2_ (ppb)	0.01 (−0.19 to 0.21)	0.04 (−0.16 to 0.24)	0.04 (−0.16 to 0.24)	−0.18 (−0.38 to 0.01)
O_3_ (ppb)	−0.09 (−0.32 to 0.13)	0.05 (−0.15 to 0.25)	−0.2 (−0.39 to 0.0)	0.01 (−0.19 to 0.21)

IQR, interquartile range; AHI, apnea–hypopnea index; ODI, oxygen desaturation index; ArI, arousal index; PM_10_, particulate matter with an aerodynamic diameter of ≤ 10 μm; PM_2.5_, particulate matter with an aerodynamic diameter of ≤ 2.5 μm; NO_2_, nitrogen dioxide; SO_2_, sulfur dioxide; O_3_, ozone.

Multivariable linear regression models were adjusted for age, sex, body mass index, temperature, and relative humidity.

*p < 0.05; **p < 0.01.

### 3.3. Association between air pollution and body water distribution

We investigated the associations between TBW, IE distribution, and exposure to air pollutants (Table 5). First, an increased IQR for PM_10_ and PM_2.5_ in the short term (1 month) led to a significant increase in TBW (PM_10_ = 0.17, 95% CI = 0.01–0.34; PM_2.5_ = 0.18, 95% CI = 0.01–0.35). An increase in PM_10_ by one IQR unit in the mid-term (3 month) led to a significant increase in TBW (PM_10_ = 0.25, 95% CI = 0.06–0.43). Similarly, an increase in PM_10_ and PM_2.5_ by one IQR unit in the short term (1 month) led to a significant increase in IE distribution (PM_10_ = 0.52, 95% CI = 0.06–0.99; PM_2.5_ = 0.62, 95% CI = 0.12–1.09). A increase in PM_10_ by one IQR unit in the mid-term (3 months) led to a significant increase in IE distribution (PM_10_ = 0.57, 95% CI = 0.04–1.1). These findings suggest that increased exposure to PM_10_ (in 1 and 3 months) and PM_2.5_ (in 1 month) results in high intracellular water content that increases IE distribution.

**Table 5:** Associations between intracellular-to-extracellular water distribution and interquartile range alterations in short-, medium-, and long-term exposure to air pollution.

Categorical variables	Beta Coefficient (95% Confidence Interval)			
	1 month	3 months	6 months	12 months
Total body water				
PM_10_ (μg/m^3^)	0.17 (0.01 to 0.34)*	0.25 (0.06 to 0.43)*	0.1 (−0.04 to 0.24)	−0.03 (−0.18 to 0.11)
PM_2.5_ (μg/m^3^)	0.18 (0.01 to 0.35)*	0.01 (−0.13 to 0.15)	0.09 (−0.05 to 0.23)	0.07 (−0.07 to 0.21)
NO_2_ (ppb)	−0.07 (−0.21 to 0.07)	0.03 (−0.11 to 0.17)	0.07 (−0.07 to 0.21)	0.07 (−0.07 to 0.21)
SO_2_ (ppb)	0.03 (−0.11 to 0.17)	−0.01 (−0.15 to 0.13)	0.09 (−0.05 to 0.23)	−0.02 (−0.16 to 0.12)
O_3_ (ppb)	−0.02 (−0.18 to 0.14)	0.05 (−0.09 to 0.19)	0.08 (−0.06 to 0.22)	0.09 (−0.05 to 0.23)
IE distribution				
PM_10_ (μg/m^3^)	0.52 (0.06 to 0.99)*	0.57 (0.04 to 1.1)*	0.39 (−0.0 to 0.79)	−0.01 (−0.41 to 0.38)
PM_2.5_ (μg/m^3^)	0.61 (0.12 to 1.09)*	0.14 (−0.26 to 0.54)	0.33 (−0.06 to 0.73)	0.13 (−0.27 to 0.53)
NO_2_ (ppb)	−0.32 (−0.72 to 0.07)	0.19 (−0.21 to 0.59)	0.33 (−0.06 to 0.73)	0.14 (−0.26 to 0.53)
SO_2_ (ppb)	0.06 (−0.34 to 0.46)	−0.19 (−0.59 to 0.21)	0.25 (−0.15 to 0.64)	−0.18 (−0.58 to 0.21)
O_3_ (ppb)	0.04 (−0.41 to 0.5)	0.08 (−0.32 to 0.47)	0.2 (−0.19 to 0.6)	0.01 (−0.39 to 0.41)

IE distribution, intracellular-to-extracellular water distribution; IQR, interquartile range; PM_10_, particulate matter with an aerodynamic diameter of ≤ 10 μm; PM_2.5_, particulate matter with an aerodynamic diameter of ≤ 2.5 μm; NO_2_, nitrogen dioxide; SO_2_, sulfur dioxide; O_3_, ozone.

Multivariable linear regression models were adjusted for age, sex, body mass index, temperature, and relative humidity; IE distribution = ICW/ECW × 100.

*p < 0.05.

### 3.4. Mediation analysis of associations between short-term air pollutants, body water ratio, and sleeping quality indices

**Figure 1:**
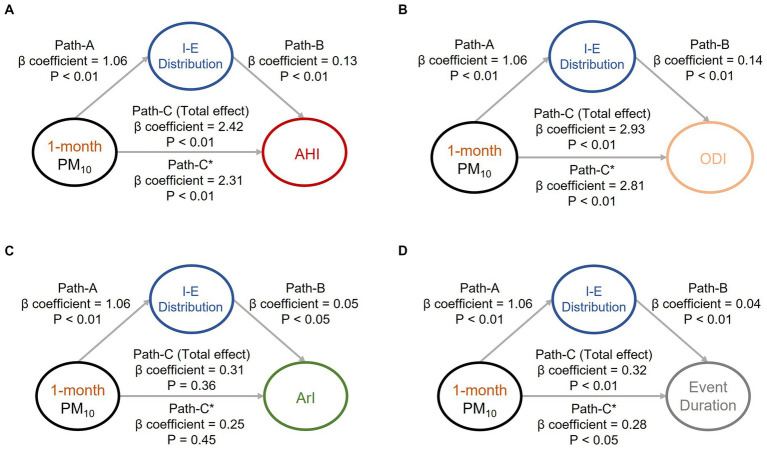
Mediation analyses of associations among intracellular-to-extracellular water distribution (IE distribution), short-term exposure (1 month) to particulate matter with an aerodynamic diameter of < 10 μm (PM_10_), and OSA severity indices. Short-term exposure (1 month) to PM_10_ was revealed to partially mediate the associations between IE distribution and OSA severity indices, including **(A)** apnea–hypopnea index, **(B)** oxygen desaturation index, **(C)** arousal index, and **(D)** respiratory event duration. The beta coefficients of the adjusted linear regression models and derived *p* values are presented in the path diagram. All four requirements for confirming a mediation effect were satisfied; that is, paths (A, B, C, and C)* were statistically significant. IE distribution, ratio of intracellular water to extracellular water; OSA, obstructive sleep apnea; PM_10_, particulate matter with an aerodynamic diameter of < 10 μm; AHI, apnea–hypopnea index; ODI, oxygen desaturation index; ArI, arousal index.

**Figure 2:**
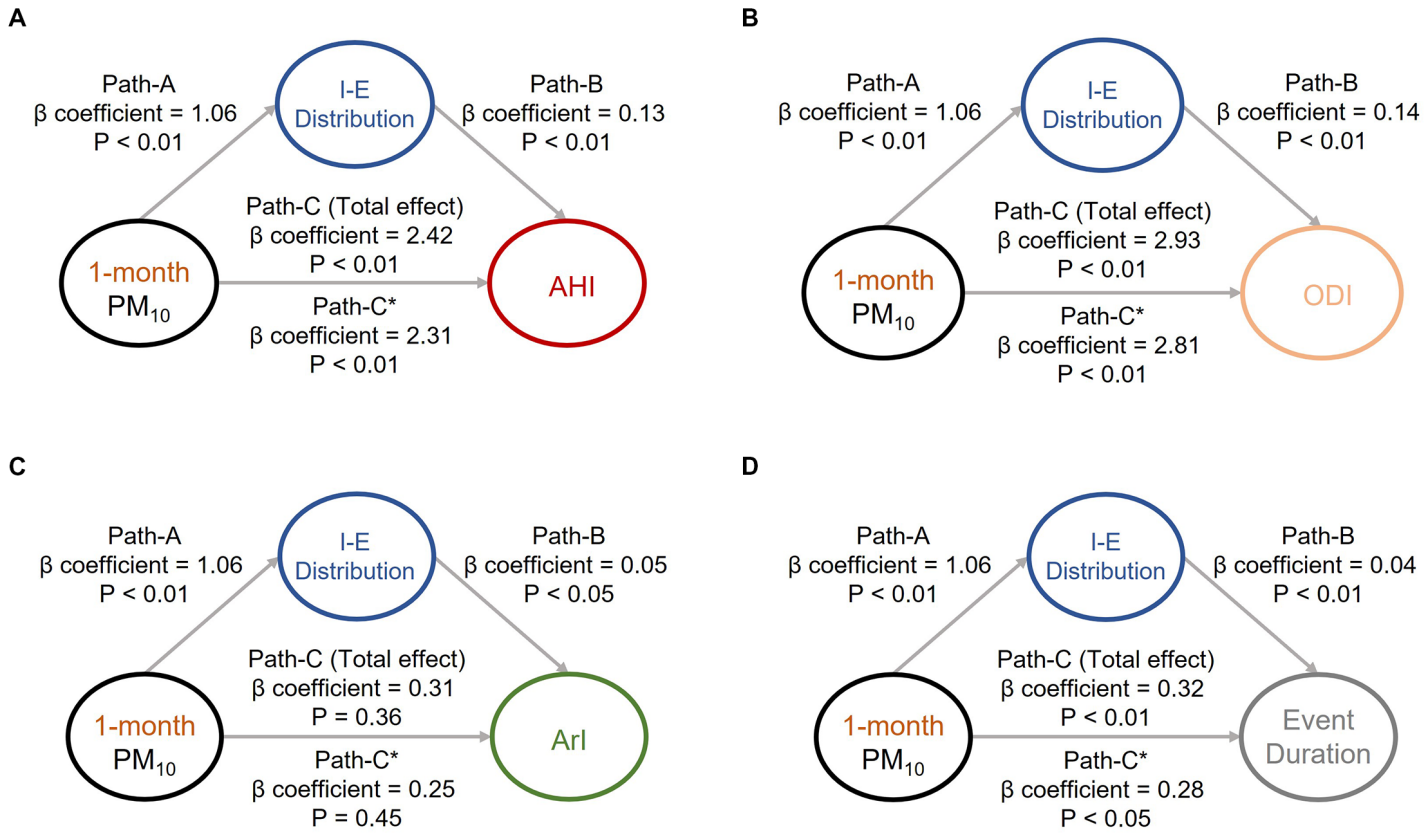
Mediation analyses of associations among intracellular-to-extracellular water distribution (IE distribution), short-term exposure (1 month) to particulate matter with an aerodynamic diameter of < 2.5 μm (PM_2.5_), and OSA severity indices. Short-term exposure (1-month) to PM_2.5_ was revealed to partially mediate the associations between IE distribution and OSA severity indices, including **(A)** apnea–hypopnea index, **(B)** oxygen desaturation index, **(C)** arousal index, and **(D)** respiratory event duration. The beta coefficients of the adjusted linear regression models and derived *p* values are presented in the path diagram. All four requirements for confirming a mediation effect were met: that is, paths A, B, C, and C* were statistically significant. IE distribution, ratio of intracellular water to extracellular water; PM_2.5_, particulate matter with an aerodynamic diameter of < 2.5 μm; AHI, apnea–hypopnea index; ODI, oxygen desaturation index; ArI, arousal index.

To investigate the causal effects pertaining to the aforementioned associations (associations of air pollution exposure with body water and OSA manifestations) that were identified, we conducted mediation effect analysis to investigate the associations among the sleep quality indices, body water distribution, and key air pollutants (i.e., short-term PM_10_ (Figure 1) and PM_2.5_ (Figure 2). Increased exposure to PM_10_ was directly associated with increases in AHI (Figure 1A), ODI, (Figure 1B) and event duration (Figure 1D) values; notably, ArI was not directly affected (Figure 1C). Similarly, increased exposure to PM_2.5_ was associated with increases in AHI (Figure 2A), ODI (Figure 2B), ArI (Figure 2C), and event duration (Figure 2D) values. These findings indicate that short-term exposure (1 month) to PM_10_ and PM_2.5_ may increase IE distribution and thereby partially intensify OSA severity or prolong event duration.

### 3.5. Association of air pollution with OSA risk

The present study analyzed the association of OSA risk with various air pollutants. As summarized in Table 6, the IQR for short-term exposure to PM_10_ (*p* < 0.05) and PM_2.5_ (*p* < 0.01) was associated with a higher risk of OSA. Specifically, an increase in the IQR for short-term exposure to PM_10_ and PM_2.5_ increased the odds ratio by 1.35 (95% CI = 1.05–1.75) and 1.42 (95% CI = 1.12–1.88). The logistic regression model results indicated that a high level of exposure to PM_10_ and PM_2.5_ in the short term (1 month) is a risk factor for OSA.

**Table 6:** Comparison of patients with and without OSA in terms of interquartile range alterations in short-, medium-, and long-term exposure to air pollution.

Categorical variables	OR (95% Confidence interval)			
	1 month	3 months	6 months	12 months
PM_10_ (μg/m^3^)	1.35 (1.05 to 1.75)*	1.14 (0.86 to 1.5)	1.1 (0.92 to 1.31)	1.04 (0.85 to 1.27)
PM_2.5_ (μg/m^3^)	1.45 (1.12 to 1.88)**	1.07 (0.89 to 1.3)	1.06 (0.86 to 1.3)	0.83 (0.7 to 1.0)
NO_2_ (ppb)	1.13 (0.94 to 1.37)	0.87 (0.72 to 1.05)	1.06 (0.87 to 1.29)	0.88 (0.74 to 1.03)
SO_2_ (ppb)	1.02 (0.89 to 1.17)	0.91 (0.8 to 1.04)	1.03 (0.9 to 1.19)	0.89 (0.76 to 1.04)
O_3_ (ppb)	1.08 (0.87 to 1.36)	0.98 (0.8 to 1.2)	0.95 (0.78 to 1.15)	1.04 (0.92 to 1.19)

OR, odds ratio; IQR, interquartile range; PM_10_, particulate matter with an aerodynamic diameter of ≤ 10 μm; PM_2.5_, particulate matter with an aerodynamic diameter of ≤ 2.5 μm; NO_2_, nitrogen dioxide; SO_2_, sulfur dioxide; O_3_, ozone.

Multivariable linear regression models were adjusted for age, sex, body mass index, temperature, and relative humidity.

*p < 0.05, **p < 0.01.

## 4. Discussion

Studies have indicated that air pollution, TBW, and body water distribution affect OSA manifestations. However, the associations between these metrics and air pollution remain unclear. Moreover, whether these metrics mediate the effects of air pollution on respiratory event duration is also unclear. The present retrospective study analyzed sleep parameters (PSG variables), anthropometric measures (body water), and estimated exposure levels to air pollutants on the basis of the residential addresses of patients, and the results reveal that short-term (1 month) exposure to PM_10_ and PM_2.5_ reduced sleep quality (i.e., AHI, ODI, and ArI) and prolonged respiratory event duration. In addition, short-term exposure to fine particles was associated with an increased risk of OSA. For body fluids, short-term (1 month) exposure to PM_10_ and PM_2.5_ was associated with increases in TBW and IE distribution. Exposure to PM_10_ and PM_2.5_ partially mediated IE distribution and indirectly intensified OSA manifestations.

We employed a novel method for estimating the level of exposure to air pollutants. Unlike other studies, which have mostly used data from one nearest station as a single exposure source. we used data from multiple stations. First, the derived estimated median level was 12.61 μg/m^3^ for PM_2.5_ and 24.77 μg/m^3^ for PM_10_, which are higher than the annual mean levels of PM_2.5_ (5 μg/m^3^) and PM_10_ (15 μg/m^3^) as specified in the 2021 air quality guidelines issued by the World Health Organization (18). In a study that employed the nearest estimation method to collect data on the air pollution in Taipei for the period from 2005 to 2012, the mean PM_10_ level was 48.3 μg/m^3^ (37.6 to 52.2 μg/m^3^), and the mean PM_2.5_ level was 27.1 μg/m^3^ (22.3 to 30.5 μg/m^3^; (19). Another study employed the nearest estimation method to estimate the extent to which people were exposed to air pollutants in Taipei for the period from 2006 to 2013, and it reported that the IQR for annual exposure to particulate pollutants was 7.3 μg/m^3^ for PM_10_ and 3.4 μg/m^3^ for PM_2.5_ (20). These estimates are higher than those reported in the present study.

The results of the present study reveal that short-term (1 month) exposure to particulate pollutants (i.e., PM_10_ and PM_2.5_) was significantly and positively associated with exacerbated OSA manifestations (i.e., increased AHI, ODI, and ArI levels as well as prolonged respiratory event duration) after adjustments for age, sex, and BMI. Several underlying mechanisms may account for this association. First, particulate pollutants may induce inflammatory responses in the pulmonary system and cause edema in the respiratory tract (21). These reactions can result in airway narrowing or collapse, which exacerbates OSA clinical manifestations. In addition, particulate pollutants may contribute to inflammatory reactions or infection in the nasal cavity and oropharyngeal tract, and these factors may induce sinusitis or rhinosinusitis and impair sleep by aggravating OSA symptoms (22). Particulate pollutants may deposit in the lower respiratory tract and cause inflammation and oxidative responses (23). Furthermore, fine particles may increase oxidative stress, which can overwhelm antioxidant defense mechanisms and increase the risk of hypoxemia (24). Exposure to particulate pollutants can affect the central nervous system by activating toxicological mechanisms, resulting in neuroinflammation and affecting the frequency of sleep arousal or the ability to respond to respiratory events (25). Exposure to particulate pollutants may aggravate OSA manifestations by prolonging the duration of respiratory events and increasing the frequency of apnea, hypopnea, oxygen desaturation, and arousal. Consistent with the findings of the present study on the association between air pollution and OSA severity, an epidemiological study reported that exposure to a high level of PM_2.5_ in the short term was associated with sleep disturbances and an increased risk of cardiovascular diseases (26). Exposure to PM_10_ was associated with increased AHI levels in patients with severe OSA during nonrapid eye movement sleep (27). Another study analyzed the data of 6,441 study participants in the United States, and it reported that exposure to PM_10_ was associated with increased AHI and oxygen desaturation levels (<90%; (28). Collectively, the aforementioned findings indicate that exposure to air pollutants affects the physiology of both the pulmonary and central nervous systems. Thus, public awareness should be enhanced to mitigate the hazardous effects of air pollutants and alleviate OSA manifestations.

For the association between air pollution and body fluids, our results demonstrate that short-term (1 month) exposure to PM_10_ and PM_2.5_ and mid-term (3 month) exposure to PM_10_ were significantly associated with increased TBW and body water distribution (IE distribution) after adjustments for age, sex, and BMI. These findings may be explained by several factors although no evidence indicates that fine particles affect body fluid retention, especially in intracellular spaces. First, exposure to particulate pollutants increases oxidative stress, which can interfere with the ion channel or gradient in the cell membrane and thereby affect osmotic pressure (29). Particulate pollutants may act as cytotoxic agents that can trigger autoimmune responses (i.e., macrophage activation), and these reactions may increase osmotic pressure and, consequently, cause systemic inflammation (30). These toxic effects of particulate pollutant exposure may increase the vascular permeability of endothelial cells and contribute to the buildup of excess intracellular fluid, resulting in cell swelling (31). A study reported that fine particles increase oxidative stress in the intracellular environment, which aggravates inflammatory reactions and causes tissue edema (32). Another possible factor is that particulate pollutants can deactivate the membrane barrier function (i.e., defense mechanisms) of the epithelium and mucosa in the airways (33). In addition, the organic components of fine particles can act as hazard factors that cause dysregulation of the cell cycle or an increase in cell inflammation, and this phenomenon can increase the osmotic pressure of the cell membrane (34). Collectively, these physiological responses to particulate pollutant exposure may partially cause body water retention and alter the body water distribution between intracellular and extracellular environments.

We conducted mediation analyses and determined that IE distribution mediated the effects of exposure to particulate pollutants (i.e., PM_2.5_ and PM_10_) on OSA manifestations. To the best of our knowledge, this is the first study to investigate the synergistic effects of air pollution, body water distribution, and OSA manifestations. Although the mechanisms underlying these synergistic effects require further elucidation, the outcomes can be interpreted on the basis of the aforementioned potential mechanisms. Fluid retention may worsen edema in airway tissues and narrow the airways, thereby aggravating OSA manifestations (35, 36). Because particulate pollutants may interfere with cell or membrane function and cause inflammatory reactions or infection (37, 38), such pollution may alter IE body water distribution. Thus, exposure to fine particles may directly aggravate OSA severity and indirectly exacerbate OSA manifestations. The present findings indicate that water distribution may be partially affected by fine particles because they can interfere with cell function and affect cell physiology, and such alterations in body water distribution may aggravate OSA manifestations or severity.

We investigated the association between exposure to air pollutants and OSA risk and discovered that exposure to air pollutants (i.e., PM_2.5_ and PM_10_) was a potential risk factor for OSA. This finding is similar to those of numerous studies, which reported a positive association between exposure to air pollution (especially PM_2.5_ and PM_10_) and OSA risk (39). An epidemiological study of 6,441 study participants indicated that exposure to particulate pollutants increased the frequency of nocturnal hypoxia and respiratory events (28). Several mechanisms may explain these outcomes. First, various studies involving animal model–based experiments have reported that fine particles aggravate the inflammatory response and increase mucus secretion in the pulmonary system, thereby causing upper airway edema or cell swelling and increasing the resistance of the respiratory tract (40). Fine particles may also induce inflammatory reactions in nasal epithelial cells and cause infections, chronic rhinosinusitis, and upper airway obstruction (41, 42). Exposure to PM_2.5_ and PM_10_ can increase oxidative stress and systemic inflammation, thereby exacerbating the occurrence of nocturnal hypoxia (43). Particulate pollutants may affect bronchial physiology, leading to an increased risk of OSA (44, 45). Therefore, exposure to particulate pollutants may be a risk factor for OSA.

The present study has several limitations that should be highlighted. First, the present study estimated the level of exposure to particulate pollutants by using data from government-maintained air quality monitoring stations. These data reflect outdoor air quality, which might differ from indoor air quality. However, the present study did not measure or estimate the level of indoor exposure to air pollutants. We employed a novel approach to overcome the limitation associated with the use of data only from a nearest station, that is, the risk of results being affected by outliers when such data are used and the nearest station is located far away from a patient’s residential address. Differences in urban land use may also affect exposure to air pollution. Given that we did not consider this factor in our present estimates, we could have underestimated the actual level of exposure. For comorbidities, although the present study excluded patients who regularly used hypnotics and those given a diagnosis of central nervous system disorders or lung diseases, other types of illnesses (e.g., cardiovascular or renal diseases) could still have affected OSA clinical manifestations (46). Moreover, the present study did not acquire information on life habits (e.g., tobacco use, alcohol use, and passive smoking) and socioeconomic status (occupation type), which are factors that could have residual confounding effects that reduce the precision of air pollution estimations or indirectly affect clinical OSA manifestations (47, 48). The sample analyzed in the present study did not fully represent the general population, and various dimensions were not considered. Thus, future studies should analyze more comprehensive data to enhance the robustness of our findings.

## 5. Conclusion

To investigate the associations among air pollutants, body water distribution, and OSA manifestations and the synergistic effects of these metrics, the present study collected data pertaining to PSG results and body composition profiles and estimated the extent to which a sample of patients were exposed to air pollutants across multiple time scales. The results revealed that a high level of short-term exposure (1 month) to fine particles, including PM_2.5_ and PM_10_, was significantly associated with increased AHI, ODI, and ArI levels and a longer respiratory event duration. Furthermore, short-term exposure to fine particulate matter affected intracellular-to-extracellular body water distribution, which partially aggravated OSA manifestations. Short-term exposure to both PM_2.5_ and PM_10_ was associated with an increased risk of OSA. Therefore, reducing the level of exposure to these fine particles may alleviate OSA severity and reduce OSA risk. The present study contributes to the literature by elucidating the potential mechanisms underlying the relationships between air pollution, body fluid parameters, and OSA severity. The identified mediating effects of air pollution on the exacerbation of OSA severity through body water distribution may be a novel basis for alleviating OSA manifestations.

## Data availability statement

The raw data supporting the conclusions of this article will be made available by the authors, without undue reservation.

## Ethics statement

The studies involving human participants were reviewed and approved by the Joint Institutional Review Board of Taipei Medical University (approval number: N202212067). Written informed consent for participation was not required for this study in accordance with the national legislation and the institutional requirements.

## Author contributions

W-TL and C-YT conceptualized and designed the study. C-YT, H-TH, and ML analyzed the data and drafted the manuscript. W-HC, W-HH, and Y-CK conducted data curation and investigation and prepared the manuscript. AM, K-YL, P-HF, and W-TL critically revised the manuscript and made essential intellectual contributions. C-HT, K-YC, and C-JW provided analytical suggestions. J-HK and H-CL administered the project. All authors contributed to the article and approved the submitted version.

## Funding

This study was funded by Taiwan’s Ministry of Science and Technology (Grant number: MOST 110–2634–F-002–049) and National Science and Technology Council (Grant number NSTC 111-2634-F-002-021). These funders had no role in the study design, data collection and analysis, decision to publish, or preparation of the manuscript.

## Conflict of interest

The authors declare that the research was conducted in the absence of any commercial or financial relationships that could be construed as a potential conflict of interest.

## Publisher’s note

All claims expressed in this article are solely those of the authors and do not necessarily represent those of their affiliated organizations, or those of the publisher, the editors and the reviewers. Any product that may be evaluated in this article, or claim that may be made by its manufacturer, is not guaranteed or endorsed by the publisher.
