# Supplementary material for: Associations between air pollution, intracellular-to-extracellular water distribution, and obstructive sleep apnea manifestations
Source: Front Public Health. 2023 Jun 15;11:1175203. doi: 10.3389/fpubh.2023.1175203 (PMC10310528; doi:10.3389/fpubh.2023.1175203)
Supplement: Supplementary file 1 [file Data_Sheet_1.PDF]

Supplementary Material

Association between air pollution, intracellular and extracellular water distribution, and obstructive sleep apnea manifestations

1 Supplementary Figures and Tables

1.1 Supplementary Figures

Supplementary Figure 1. Adjusted approach for exposure estimation.

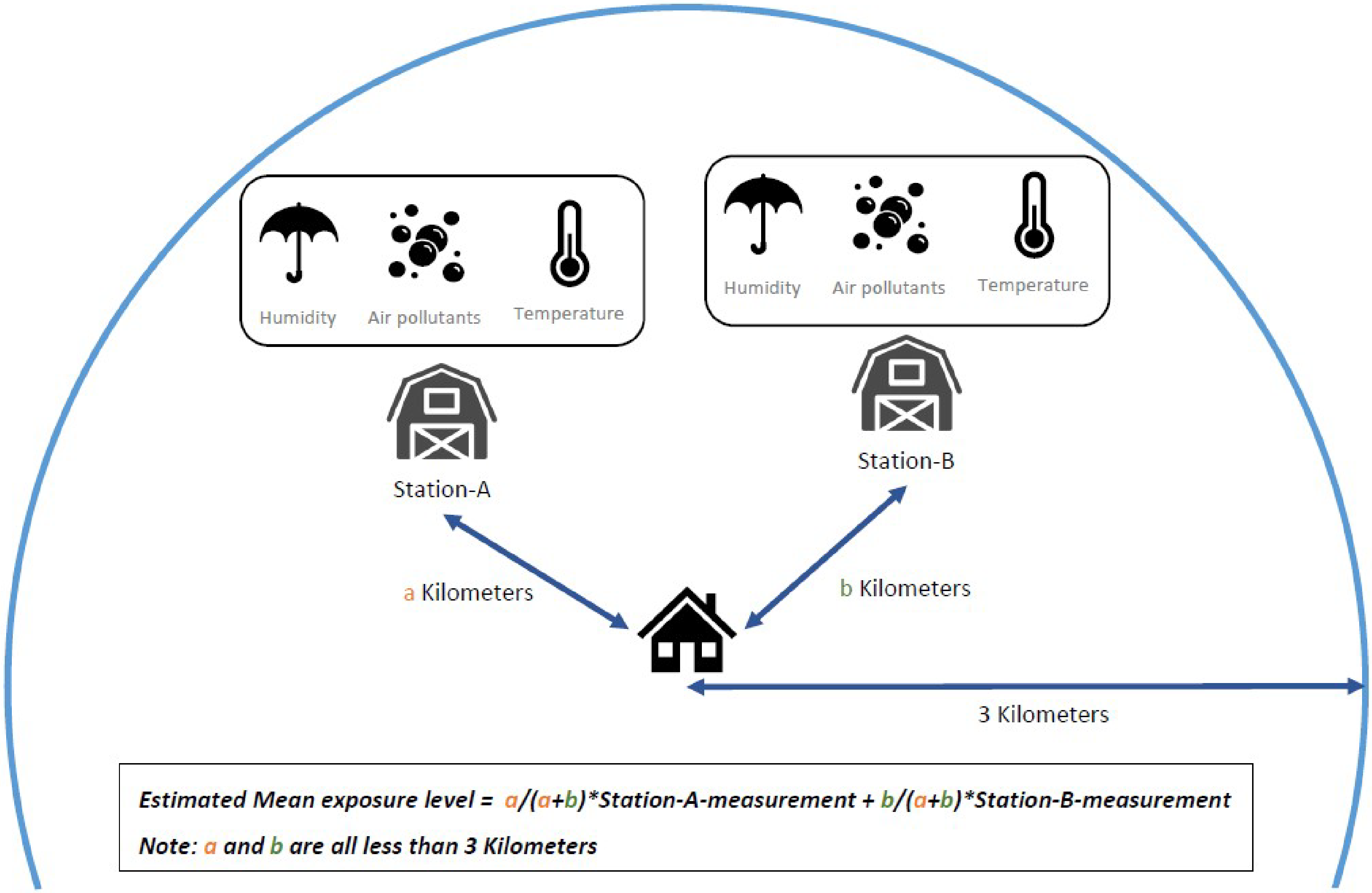

With reference to the approach that employed the measurements of the nearest station as exposure estimation, this study adjusted the approach by using weighted distribution and also limited the distance. By utilizing these, we could avoid scenarios where the nearest station was still far, which would have resulted in acquiring unreliable estimations.

1.2 Supplementary Tables

Table S1. Associations between sleep quality indices and an interquartile range (IQR) alteration in short-, medium-, and long-term exposure to air pollution in males (N=1926).

Categorical variables	Beta Coefficient (95% Confidence Interval)			
	1 month	3 months	6 months	12 months
**AHI** (events/h)				
PM_{10} (μg/m^3)	2.06 (0.97 to 3.15) **	-0.56 (-1.8 to 0.68)	0.86 (-0.1 to 1.81)	-0.39 (-1.35 to 0.57)
$PM_{2.5}$ (μg/m^3)	3.35 (2.2 to 4.5) **	0.03 (-0.92 to 0.99)	0.8 (-0.15 to 1.75)	0.44 (-0.52 to 1.39)
NO_2 (ppb)	0.32 (-0.63 to 1.28)	-1.18 (-2.13 to -0.23)	0.46 (-0.49 to 1.42)	-0.06 (-1.01 to 0.9)
SO_2 (ppb)	-0.27 (-1.22 to 0.69)	0.71 (-0.25 to 1.66)	0.18 (-0.78 to 1.13)	-0.94 (-1.89 to 0.02)
O_3 (ppb)	0.98 (-0.11 to 2.06)	0.25 (-0.7 to 1.21)	1.14 (0.18 to 2.09)	0.42 (-0.53 to 1.38)
**ODI** (events/h)				
PM_{10} (μg/m^3)	2.16 (1.07 to 3.25) **	-0.3 (-1.55 to 0.94)	0.64 (-0.31 to 1.6)	-0.16 (-1.12 to 0.8)
$PM_{2.5}$ (μg/m^3)	3.75 (2.59 to 4.9) **	-0.19 (-1.15 to 0.76)	0.62 (-0.33 to 1.58)	0.55 (-0.4 to 1.51)
NO_2 (ppb)	0.17 (-0.78 to 1.13)	-1.21 (-2.17 to -0.26)	0.3 (-0.66 to 1.26)	0.18 (-0.78 to 1.14)
SO_2 (ppb)	-0.48 (-1.44 to 0.48)	0.78 (-0.18 to 1.73)	-0.02 (-0.98 to 0.93)	-0.95 (-1.91 to 0.0)
O_3 (ppb)	0.92 (-0.17 to 2.01)	0.13 (-0.82 to 1.09)	0.89 (-0.07 to 1.84)	0.18 (-0.77 to 1.14)
**ArI** (events/h)				
PM_{10} (μg/m^3)	1.18 (0.41 to 1.95) **	-0.73 (-1.59 to 0.14)	0.81 (0.14 to 1.47)	-0.23 (-0.9 to 0.43)
$PM_{2.5}$ (μg/m^3)	1.67 (0.86 to 2.49) **	0.13 (-0.54 to 0.79)	0.86 (0.19 to 1.52)	-0.24 (-0.91 to 0.42)
NO_2 (ppb)	-0.15 (-0.83 to 0.52)	-0.83 (-1.49 to -0.16)	0.71 (0.04 to 1.37)	-0.31 (-0.97 to 0.36)

SO_2 (ppb)	-0.07 (-0.74 to 0.6)	0.59 (-0.08 to 1.26)	0.21 (-0.45 to 0.88)	-0.12 (-0.78 to 0.55)
O_3 (ppb)	-0.43 (-1.2 to 0.33)	0.42 (-0.25 to 1.09)	0.71 (0.04 to 1.37)	0.24 (-0.42 to 0.91)
**Respiratory event duration** (s)				
PM_{10} (μg/m^3)	0.24 (-0.04 to 0.53)	0.07 (-0.25 to 0.39)	-0.14 (-0.38 to 0.11)	0.01 (-0.24 to 0.26)
$PM_{2.5}$ (μg/m^3)	1.13 (0.83 to 1.43) **	-0.07 (-0.32 to 0.17)	-0.16 (-0.4 to 0.09)	-0.18 (-0.43 to 0.06)
NO_2 (ppb)	0.08 (-0.17 to 0.33)	-0.01 (-0.26 to 0.24)	-0.16 (-0.4 to 0.09)	-0.12 (-0.36 to 0.13)
SO_2 (ppb)	-0.01 (-0.25 to 0.24)	0.04 (-0.2 to 0.29)	-0.12 (-0.37 to 0.12)	-0.27 (-0.51 to -0.02)
O_3 (ppb)	-0.14 (-0.42 to 0.14)	-0.06 (-0.3 to 0.19)	-0.18 (-0.43 to 0.07)	0.09 (-0.15 to 0.34)

Abbreviations: AHI: apnea–hypopnea index; ODI*:* oxygen desaturation index; ArI: arousal index; PM_{10}, particulate matter with an aerodynamic diameter of ≤10 μm; $PM_{2.5}$, particulate matter with an aerodynamic diameter of ≤2.5 μm; NO_2, nitrogen dioxide; SO_2, sulfur dioxide; O_3, ozone.

Note: Multivariable linear regression models were adjusted for age, body mass index, temperature, and relative humidity.

* $p < 0.05$; ** $p < 0.01$.

Table S2. Associations between sleep quality indices and an interquartile range (IQR) alteration in short-, medium-, and long-term exposure to air pollution in females (N=980).

Categorical variables	Beta Coefficient (95% Confidence Interval)			
	1 month	3 months	6 months	12 months
**AHI** (events/h)				
PM_{10} (μg/m^3)	1.05 (-0.35 to 2.45) *	0.08 (-1.41 to 1.56)	-0.62 (-1.7 to 0.46)	-0.19 (-1.27 to 0.89)
$PM_{2.5}$ (μg/m^3)	1.78 (0.44 to 3.12)	0.76 (-0.32 to 1.84)	-0.83 (-1.91 to 0.25)	-0.93 (-2.0 to 0.15)
NO_2 (ppb)	-0.45 (-1.53 to 0.63)	0.56 (-0.52 to 1.64)	-0.86 (-1.93 to 0.22)	-1.01 (-2.08 to 0.07)
SO_2 (ppb)	-0.11 (-1.19 to 0.97)	-0.27 (-1.35 to 0.82)	0.06 (-1.01 to 1.14)	0.09 (-0.99 to 1.17)
O_3 (ppb)	-0.14 (-1.4 to 1.11)	-0.36 (-1.43 to 0.72)	-0.95 (-2.02 to 0.12)	0.13 (-0.95 to 1.21)
**ODI** (events/h)				
PM_{10} (μg/m^3)	1.07 (-0.25 to 2.39)	0.41 (-1.0 to 1.81)	-0.64 (-1.66 to 0.37)	0.15 (-0.87 to 1.17)
$PM_{2.5}$ (μg/m^3)	1.81 (0.55 to 3.08) *	1.04 (0.02 to 2.06)	-0.81 (-1.83 to 0.21)	-0.65 (-1.66 to 0.37)
NO_2 (ppb)	-0.37 (-1.39 to 0.65)	0.72 (-0.3 to 1.73)	-0.81 (-1.83 to 0.2)	-0.63 (-1.65 to 0.39)
SO_2 (ppb)	-0.41 (-1.43 to 0.6)	-0.07 (-1.09 to 0.95)	-0.06 (-1.07 to 0.96)	0.21 (-0.81 to 1.23)
O_3 (ppb)	0.01 (-1.17 to 1.2)	-0.2 (-1.22 to 0.81)	-0.95 (-1.96 to 0.07)	0.09 (-0.93 to 1.1)
**ArI** (events/h)				
PM_{10} (μg/m^3)	0.03 (-0.92 to 0.98)	-0.49 (-1.49 to 0.51)	-0.32 (-1.05 to 0.4)	-0.38 (-1.11 to 0.35)
$PM_{2.5}$ (μg/m^3)	0.18 (-0.74 to 1.09)	-0.05 (-0.78 to 0.68)	-0.45 (-1.17 to 0.28)	0.06 (-0.67 to 0.79)
NO_2 (ppb)	-0.02 (-0.75 to 0.72)	0.28 (-0.45 to 1.0)	-0.81 (-1.53 to -0.09)	-0.13 (-0.87 to 0.6)
SO_2 (ppb)	-0.14 (-0.88 to 0.59)	0.01 (-0.72 to 0.74)	-0.01 (-0.73 to 0.72)	0.46 (-0.28 to 1.19)
O_3 (ppb)	-0.74 (-1.59 to 0.11)	-0.29 (-1.01 to 0.44)	-0.25 (-0.97 to 0.48)	-0.15 (-0.89 to 0.58)

**Respiratory event duration** (s)				
PM_{10} ($\mu g/m^3$)	0.63 (0.2 to 1.07) **	0.1 (-0.37 to 0.56)	-0.01 (-0.34 to 0.33)	0.09 (-0.24 to 0.43)
$PM_{2.5}$ ($\mu g/m^3$)	1.31 (0.9 to 1.73) **	-0.17 (-0.51 to 0.17)	-0.01 (-0.35 to 0.33)	-0.18 (-0.52 to 0.16)
NO_2 (ppb)	-0.14 (-0.48 to 0.2)	-0.09 (-0.43 to 0.25)	0.02 (-0.32 to 0.36)	-0.13 (-0.47 to 0.21)
SO_2 (ppb)	0.03 (-0.31 to 0.37)	-0.0 (-0.34 to 0.34)	0.33 (-0.0 to 0.67)	-0.02 (-0.35 to 0.32)
O_3 (ppb)	0.02 (-0.38 to 0.41)	0.25 (-0.08 to 0.59)	-0.22 (-0.55 to 0.12)	-0.16 (-0.5 to 0.18)

Abbreviations: AHI: apnea–hypopnea index; ODI*:* oxygen desaturation index; ArI: arousal index; PM_{10}, particulate matter with an aerodynamic diameter of ≤10 μm; $PM_{2.5}$, particulate matter with an aerodynamic diameter of ≤2.5 μm; NO_2, nitrogen dioxide; SO_2, sulfur dioxide; O_3, ozone.

Note: Multivariable linear regression models were adjusted for age, body mass index, temperature, and relative humidity.

* $p < 0.05$; ** $p < 0.01$.
